# Supplementary material for: Torsional periodic lattice distortions and diffraction of twisted 2D materials
Source: Nat Commun. 2022 Dec 19;13:7826. doi: 10.1038/s41467-022-35477-x (PMC9763474; doi:10.1038/s41467-022-35477-x)
Supplement: Supplementary file 2 — Supplementary Information for Torsional Periodic Lattice Distortions and Diffraction of Twisted 2D Materials [file 41467_2022_35477_MOESM2_ESM.pdf]

Supplementary Information for

Torsional Periodic Lattice Distortions and Diffraction of Twisted 2D Materials

Suk Hyun Sung, Yin Min Goh, Hyobin Yoo, Rebecca Engelke, Hongchao Xie, Kuan Zhang, Zidong Li, Andrew Ye, Parag B. Deotare, Ellad B. Tadmor, Andrew J. Mannix, Jiwoong Park, Liuyan Zhao, Philip Kim, and Robert Hovden

Contents

Supplementary Figures

Supplementary Figure S1 Transversity of PLD . . . 2
Supplementary Figure S2 Quantification of Torsional PLD Amplitude A_1 . . . 3
Supplementary Figure S3 Contribution of PLD Harmonics across Twist Angles . . . 4
Supplementary Figure S4 Exponential Decay of PLD Harmonics . . . 5
Supplementary Figure S5 Interlayer Registry Energy of TBG with torsional PLD . . . 6
Supplementary Figure S6 Elastic Energy of torsional PLD . . . 7
Supplementary Figure S7 Local Rotational Fields of Torsional PLD . . . 8
Supplementary Figure S8 Relative Phases of torsional PLD . . . 9
Supplementary Figure S9 Lattice reconstruction in 2L+2L CrI_3 . . . 10
Supplementary Figure S10 Periodic relaxations in lattice mismatched twisted bilayer systems . . . 11
Supplementary Figure S11 Torsional PLDs in 4L-WS_2 . . . 12
Supplementary Figure S12 SAED of WSe_2/$MoSe_2$ Heterostructure . . . 13

Supplementary Notes

Supplementary Note 1 Definitions: Twisted Bilayer Graphene and its torsional PLD . . . 14
Supplementary Note 2 Derivation: Local Rotation of Torsional PLD . . . 15
2.1 Geometric Upper Bound for PLD Amplitude (A_1) . . . 16
Supplementary Note 3 Derivation: Stress, Strain and Elastic Energy of Torsional PLD . . . 17
3.1 Stiffness Tensor, Stress and Elastic Energy . . . 17
3.2 Elastic Energy per Unit Cell . . . 18

Supplementary Figure S1 Transversity of PLD

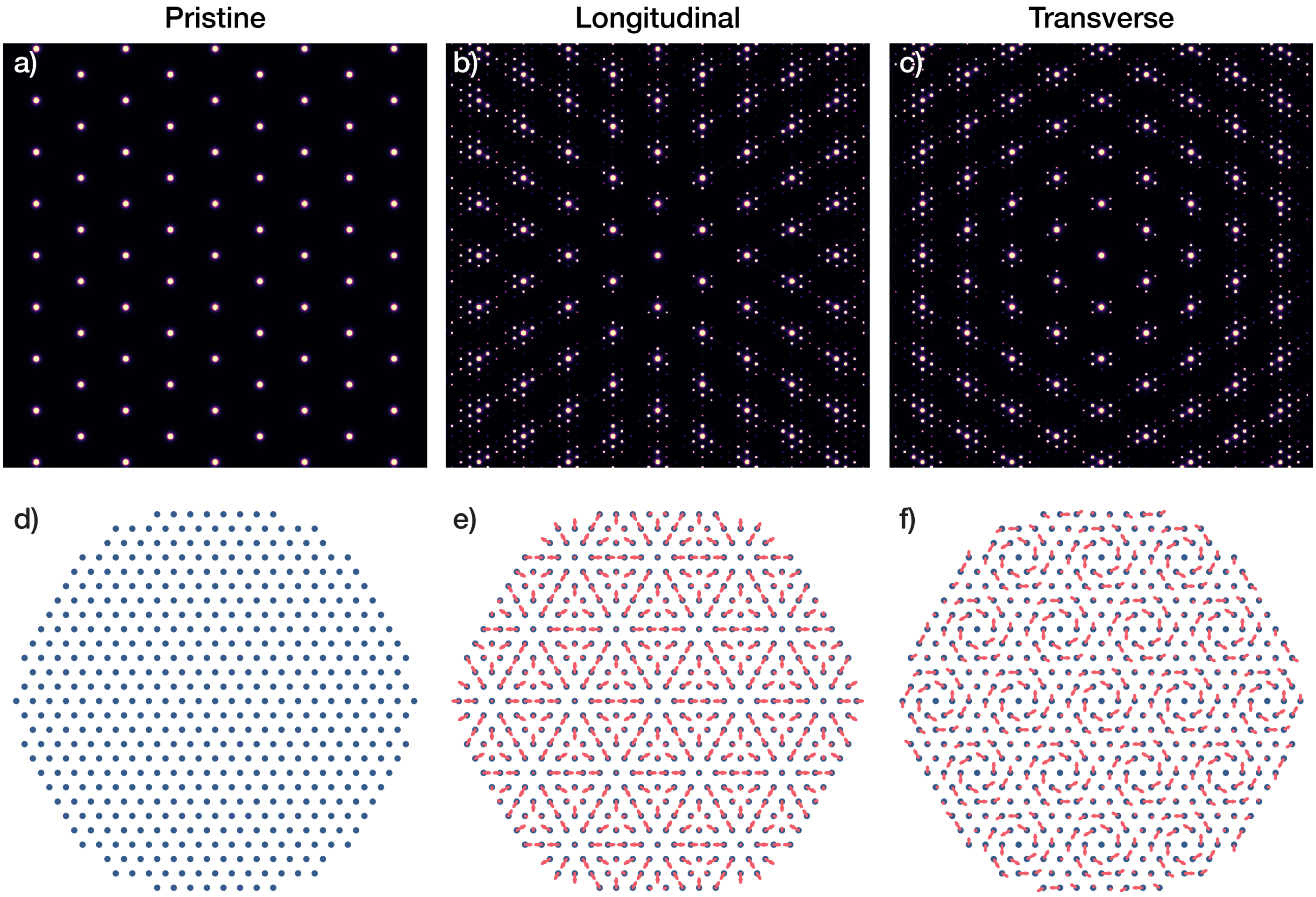

Fig.S1 | Transversity of PLD Simulated diffraction of crystal with a) no PLD, b) longitudinal PLD and c) transverse PLD. $|q|$ is set to be $5a_0$ where a_0 is pristine lattice constant ($a_0 = 5$Å, $|\mathbf{q}| = 5a_0$). Each PLD consists of three PLD waves oriented 120° apart. a) with no PLD, only the usual crystal Bragg peaks are present. b, c) superlattice peaks emerge with PLD. PLD superlattice peaks are located $\alpha\mathbf{q}$ away from Bragg peaks with intensity proportional to $|\mathrm{J}_\alpha(\mathbf{k}\cdot\mathbf{A})|^2$. Note the difference in distribution of superlattice peaks in b and c due to transversity of the PLD. d, e, f) Schematic real space diagram of pristine, longitudinal and transverse PLD. Blue circles represents pristine lattice points and red arrows denote resultant lattice distortion due to PLDs.

Supplementary Figure S2 Quantification of Torsional PLD Amplitude A_1

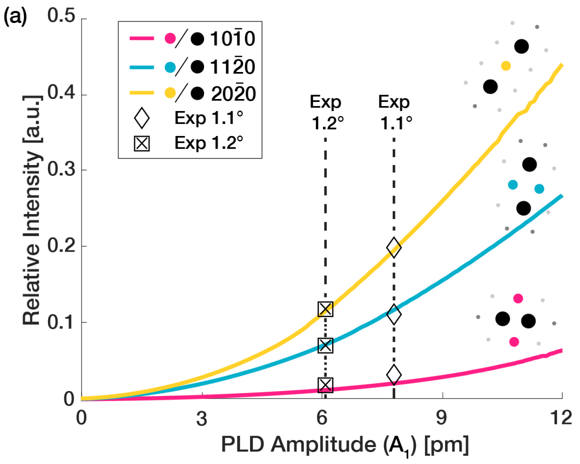

Fig.S2 | Quantification of Torsional PLD Amplitude A_1 (a) Relative intensities of superlattice peaks to Bragg peaks of first order (red), second order (blue), and third order (yellow) diffraction spots characterized over a range of PLD amplitudes. The plot reveals that 1.1° and 1.2° rTBG are relaxed by single harmonic torsional PLD with amplitude 7.8 pm and 6.1 pm, respectively.

Supplementary Figure S3 Contribution of PLD Harmonics across Twist Angles

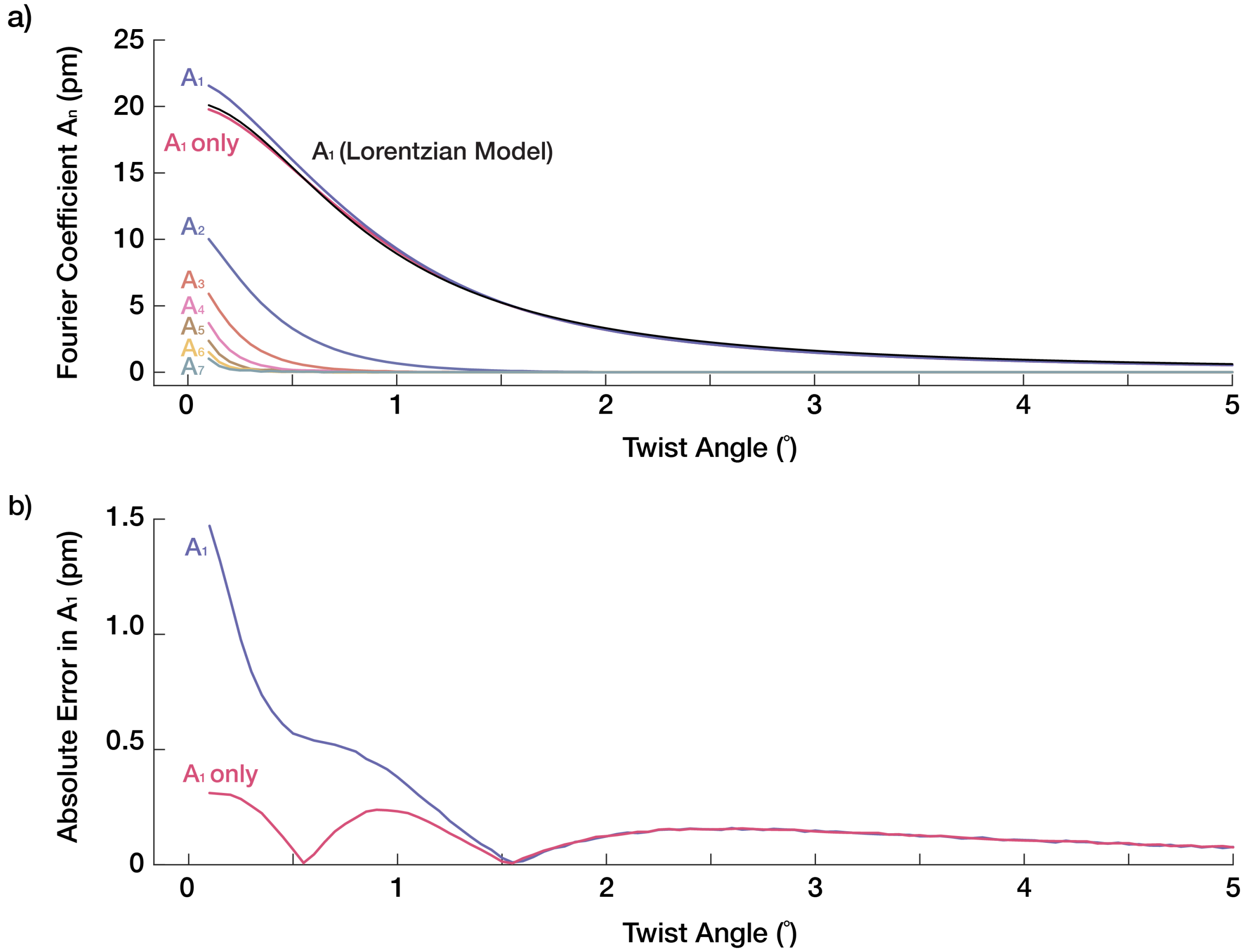

Fig.S3 | Contribution of PLD Harmonics across Twist Angles a) Amplitude of nth order PLD harmonics (A_n) against twist angle. PLD amplitudes are obtained by minimizing total energy $(V_{vdW} + V_{El})$ with up to 9th order harmonic PLD included. At higher twist angle A_1 is dominant, and A_2, A_3, A_4 exceed 1 pm at $\theta \lesssim 0.9°$, 0.45°, 0.3° respectively. Magenta 'A_1 only' was obtained by minimizing the total energy with A_1 included. A_1 traces the Lorentzian model well at high θ and slightly deviates at $\theta \lesssim 0.5°$. b) Absolute error of Lorentzian model with respect to A_1 (blue) and 'A_1 only' (red).

Supplementary Figure S4 Exponential Decay of PLD Harmonics

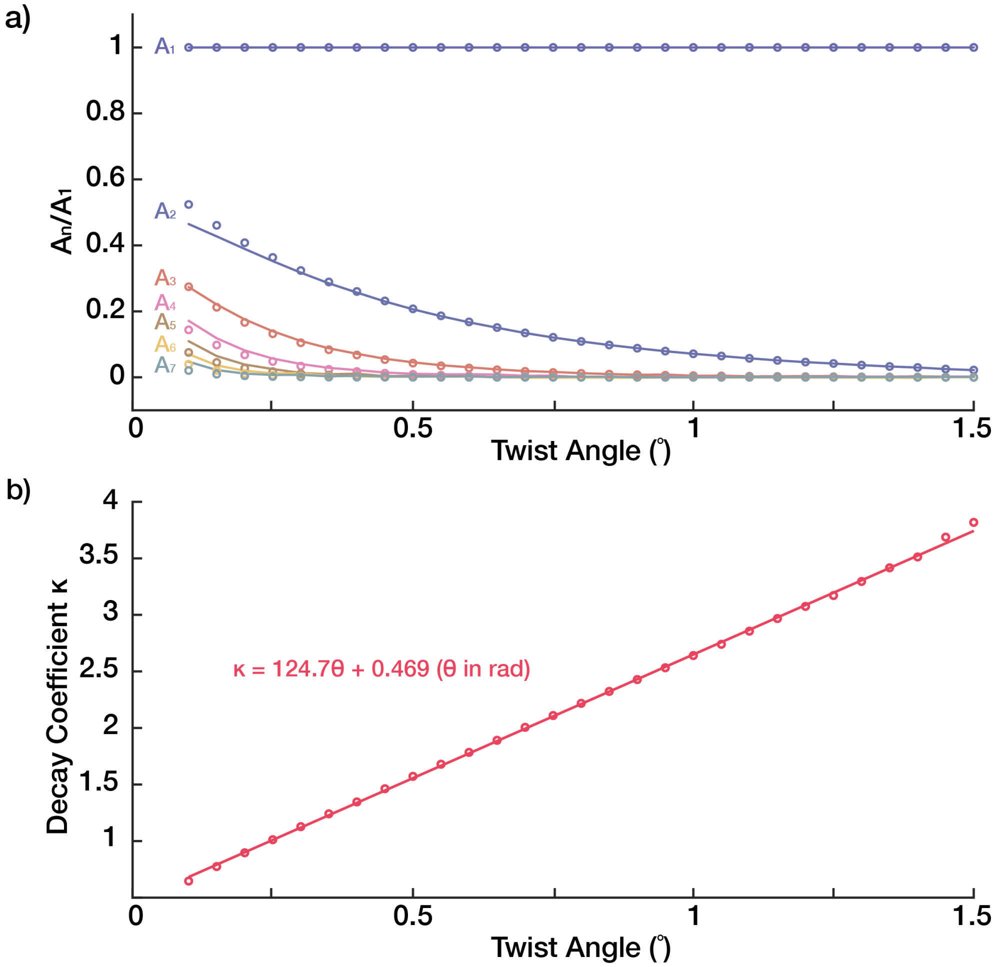

Fig.S4 | Exponential Decay of PLD Harmonics a) Relative amplitude of PLD harmonics to fundamental harmonic (A_n/A_1) plotted versus twist angle (circles) closely follows exponential decay ($A_n = A_1 e^{-\kappa(\theta)\cdot(n-1)}$) (line). b) Decay parameter, κ, is linear proportional to θ. Therefore, decreasing θ slows down decay of A_n. Notably, exponential decay is the upper bound for Fourier coefficients (Paley-Wiener theorem).

Supplementary Figure S5 Interlayer Registry Energy of TBG with torsional PLD

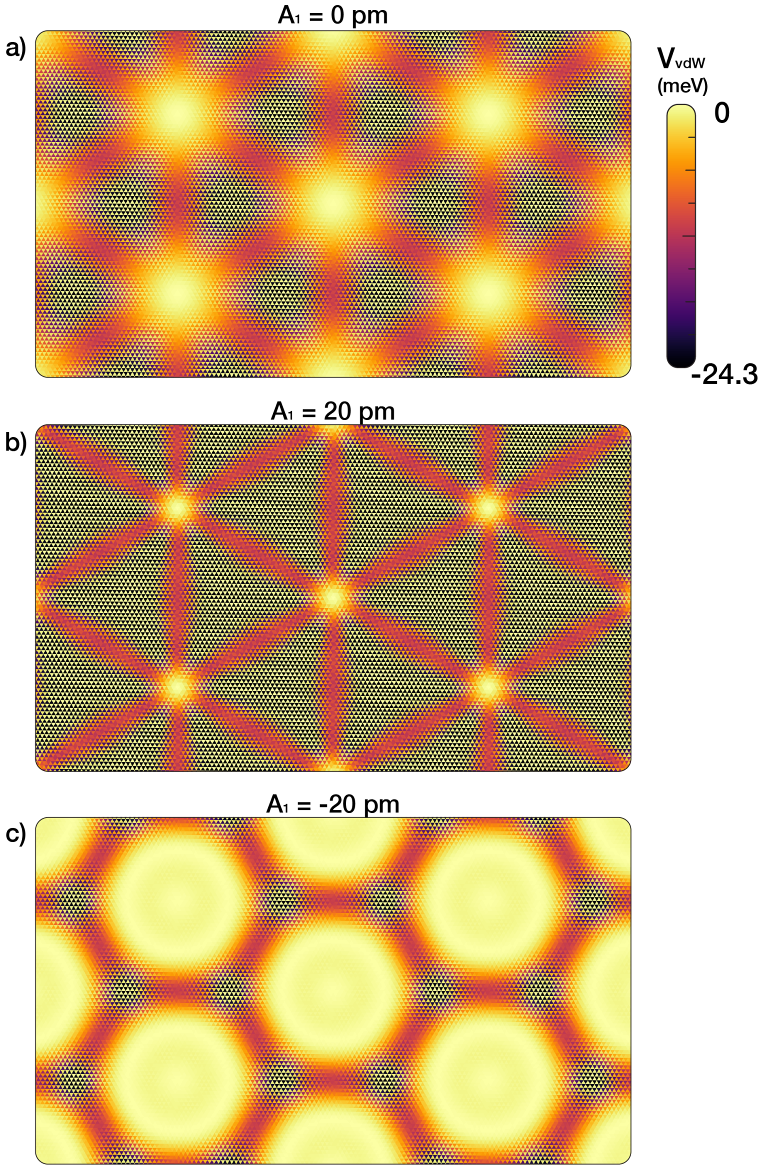

Fig.S5 | Interlayer Registry Energy of TBG with torsional PLD Interlayer vdW energy overlaid as color intensities over real-space lattice of TBG with a) no PLD, b) A_1 = 20 pm, and c) A_1 = -20 pm. b) depicts near-optimal torsional PLD that minimizes the interlayer energy without elastic cost. Note unfavorable AA-region (bright circular regions) are minimized compared to no PLD case. c) depicts worst-case scenario where AA-region is maximized. The interlayer interaction energies are calculated using Kolmogorov-Crespi potentials [1].

Supplementary Figure S6 Elastic Energy of torsional PLD

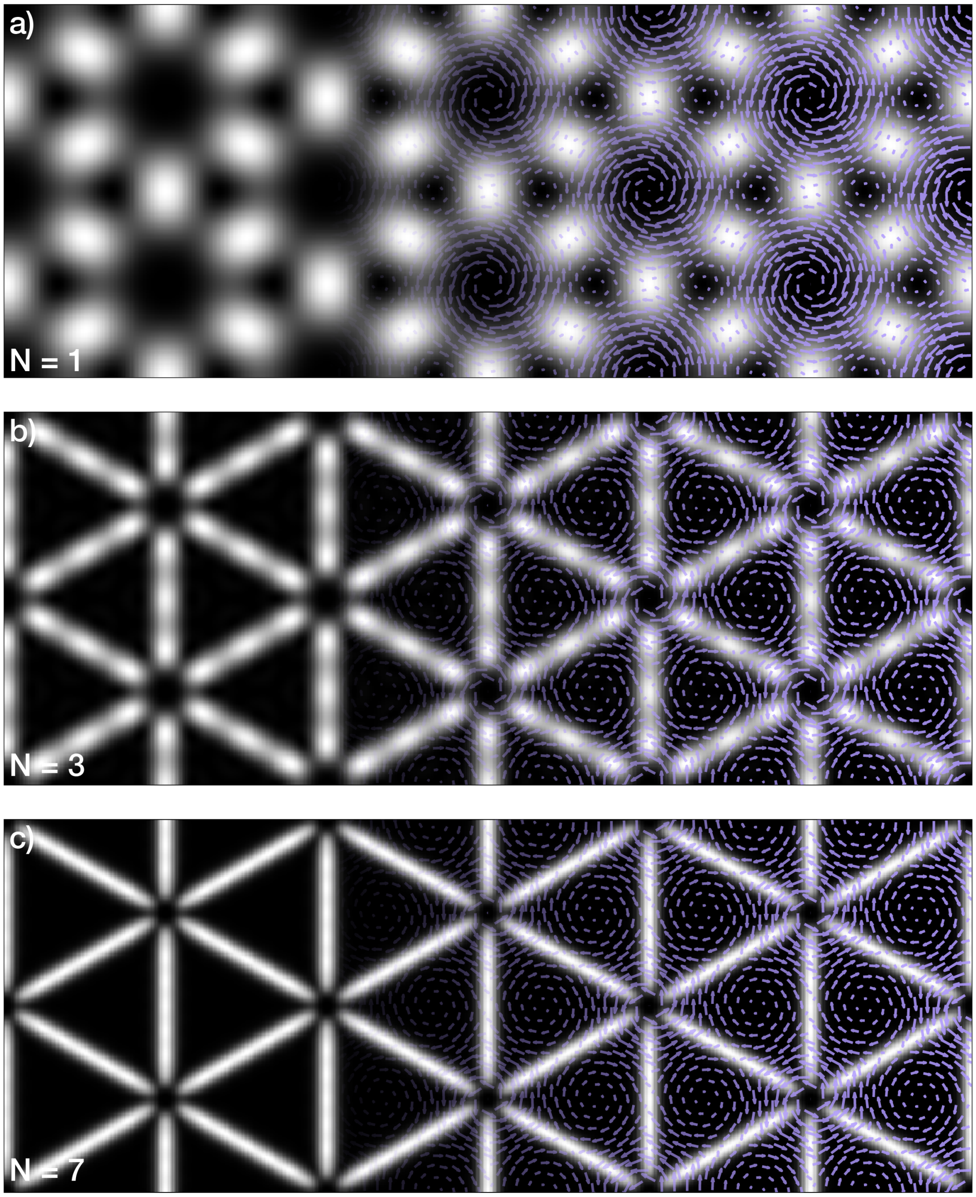

Fig.S6 | Elastic Energy of Torsional PLD V_{El} with number of harmonics a) $N = 1$, b) $N = 3$, and c) $N = 7$. Torsional PLD amplitudes of higher orders are defined as $A_n = A_1 e^{-\kappa n}$. Notably, the elastic energy of AB, BA and AA cores are lowered with PLD distortions. Similar to Ω_{N}, inclusion of higher order Fourier coefficients allow for sharper features in $N = 7$ than in $N = 1, 3$. Overlaid arrows represent torsional PLD displacement fields, Δ_{N}. Analytical derivations can be found in Supplementary Note 3.

Supplementary Figure S7 Local Rotational Fields of Torsional PLD

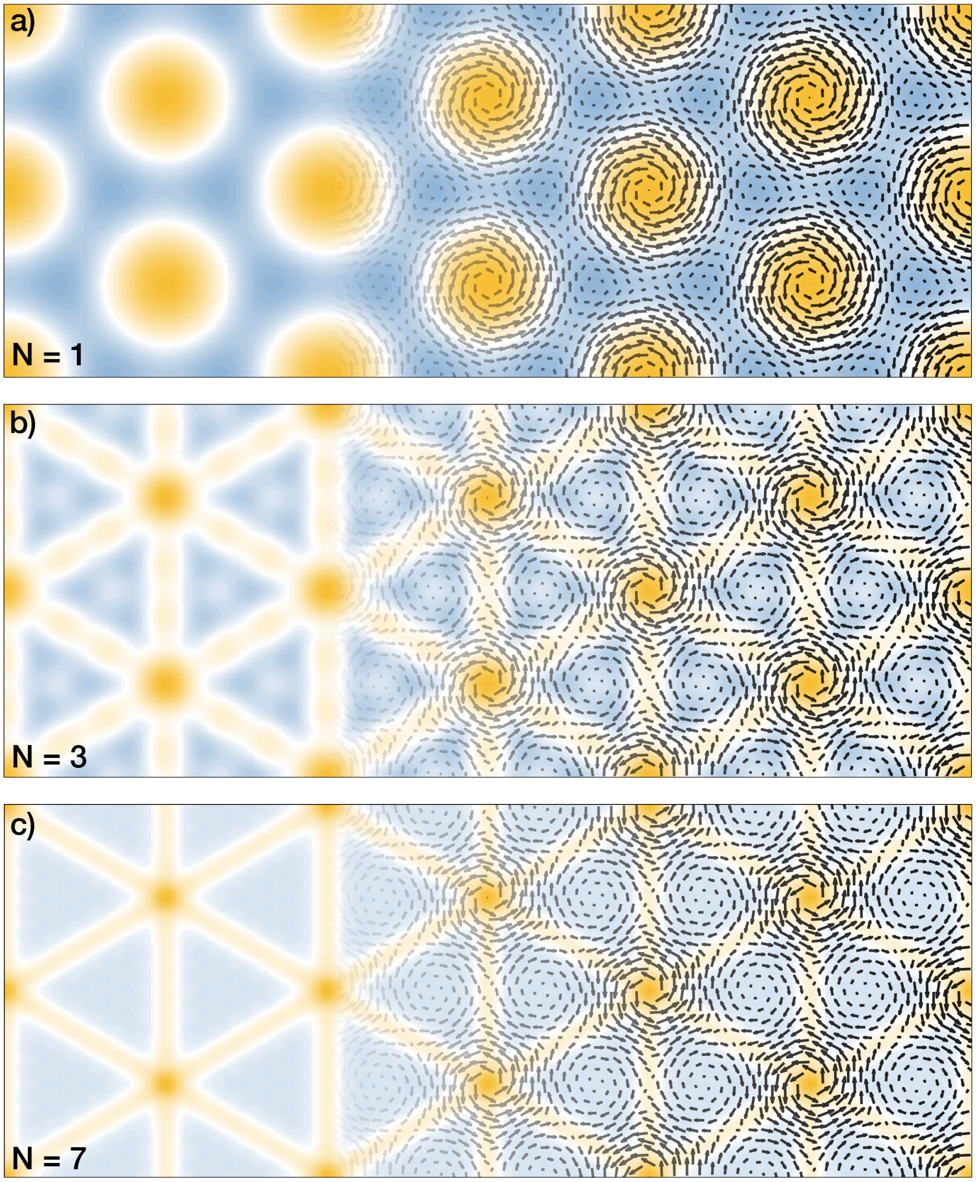

Fig.S7 | Local Rotational Fields of Torsional PLD Ω_{N} with a) $N = 1$, b) $N = 3$, and c) $N = 7$. Torsional PLD amplitudes are defined as $A_n = A_1 e^{-\kappa n}$. Inclusion of higher order Fourier coefficients allow more sharper features in $N = 7$ than in $N = 1, 3$. Overlaid arrows represents torsional PLD displacement fields Δ_{N}. Analytical derivations can be found in Supplementary Note 2.

Supplementary Figure S8   Relative Phases of torsional PLD

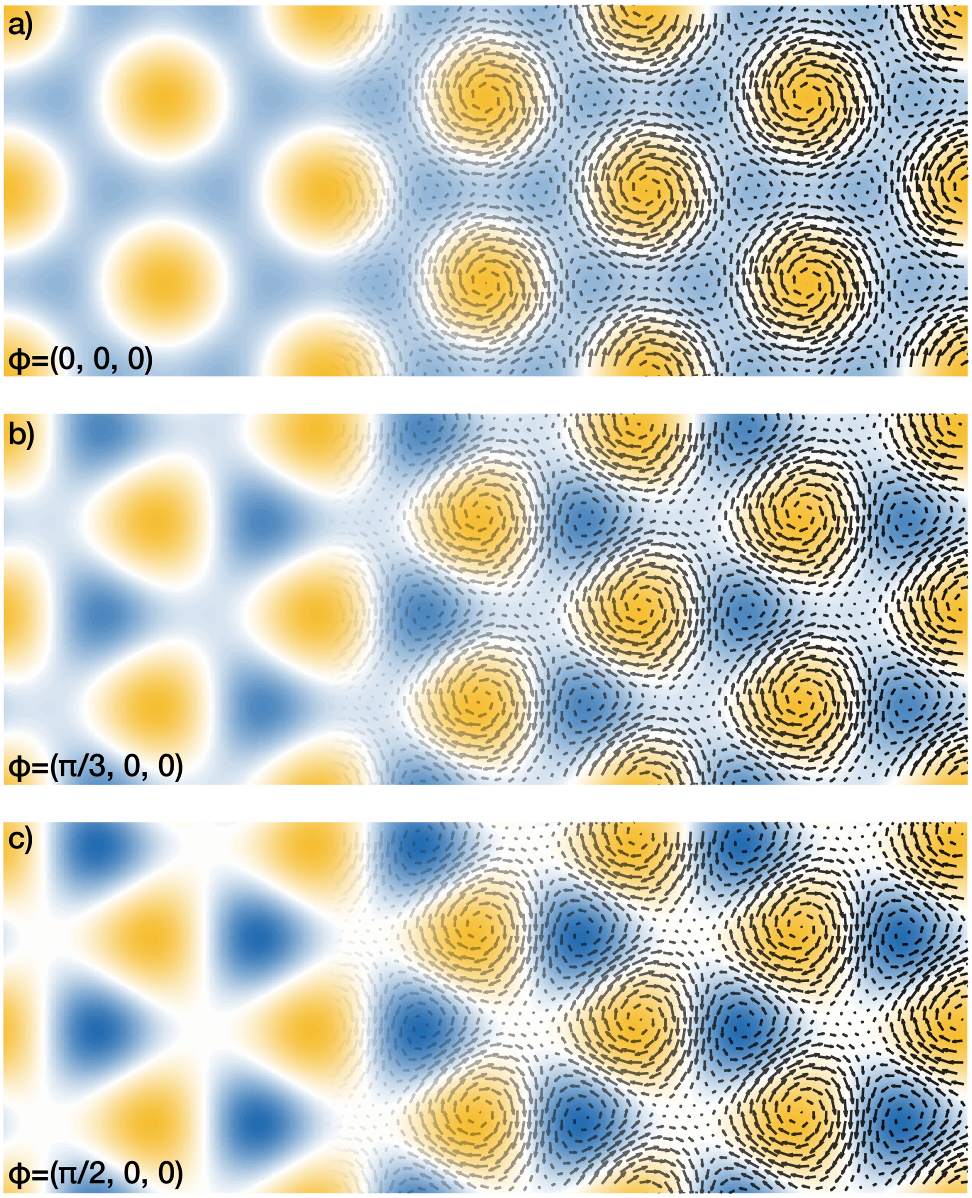

Fig.S8 | Relative Phases of torsional PLD Torsional PLD behavior can be controlled by changing the phases (ϕ_i) of constituent sinusoids. Here, rotational field (Ω_1) with $(\phi_1, \phi_2, \phi_3) =$ a) $(0, 0, 0)$, b) $(\pi/3, 0, 0)$, c) $(\pi/2, 0, 0)$ are shown.

Supplementary Figure S9 Lattice reconstruction in 2L+2L CrI_3

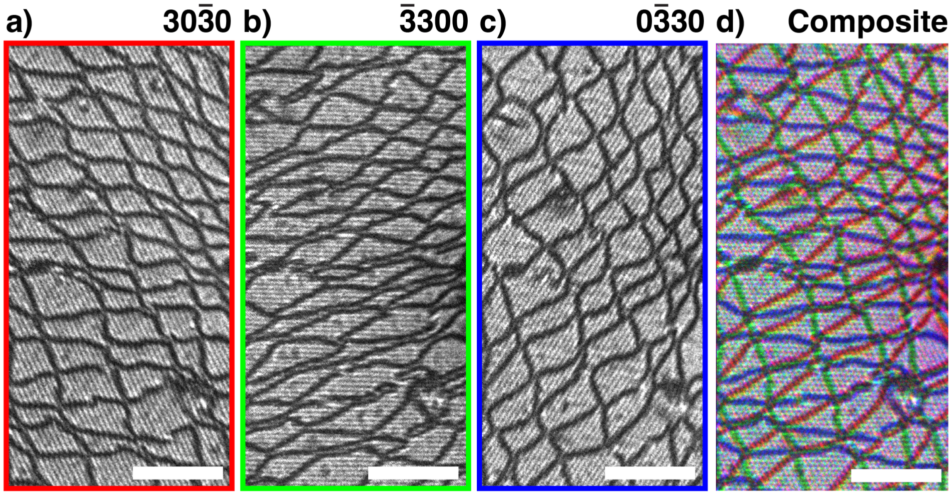

Fig.S9 | Lattice reconstruction in 2L+2L CrI_3 a–d) DF-TEM micrographs of CrI_3 taken from $\{30\bar{3}0\}$ reflections and RGB color composite from a–c. DF-TEM shows triangular domains—definitive signatures of lattice reconstruction [2]. Scale bars are 50 nm.

Supplementary Figure S10 Periodic relaxations in lattice mismatched twisted bilayer systems

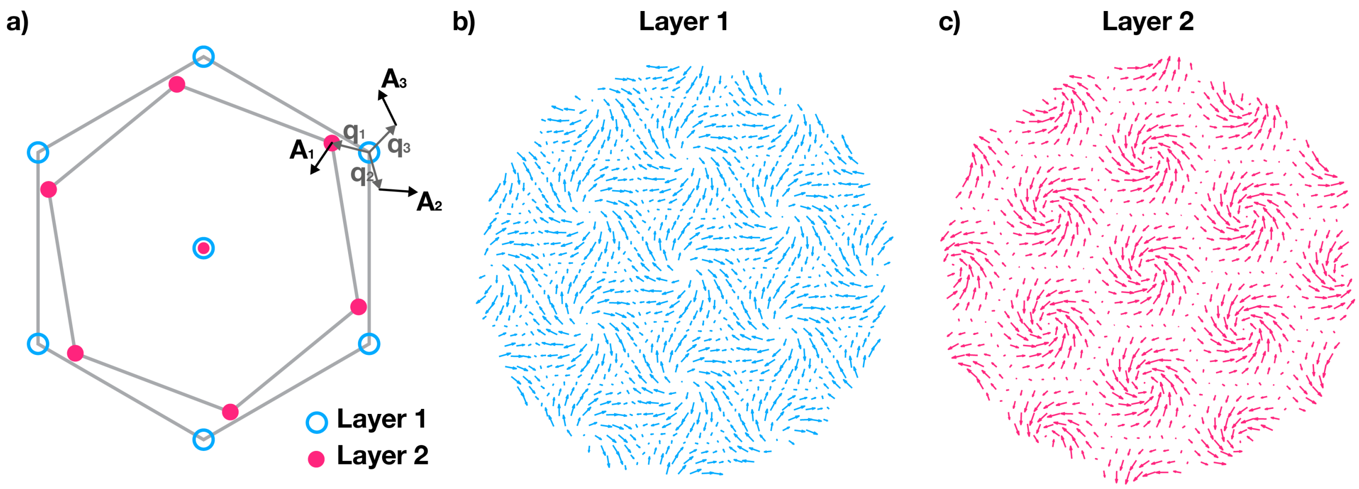

Fig.S10 | Periodic relaxations in lattice mismatched twisted bilayer systems a) Schematic reciprocal lattice structure of twisted heterostructure of two lattice constant mismatched lattice. Layer 1 has smaller real space lattice constant. Vectors connecting first order Bragg peaks ($\mathbf{q}_{1,2,3}$) defines moiré unit cell and torsional PLD wavevectors. PLD displacement vectors ($\mathbf{A}_i$'s) are chosen to be non-orthogonal to $\mathbf{q}_i$'s. b, c) PLD displacement pattern with $\angle \mathbf{q}_i \mathbf{A}_i = 45°$. Layer 1 and 2 experiences expansion and compression of lattice, respectively, as well as torsional field.

Supplementary Figure S11   Torsional PLDs in 4L-WS_2

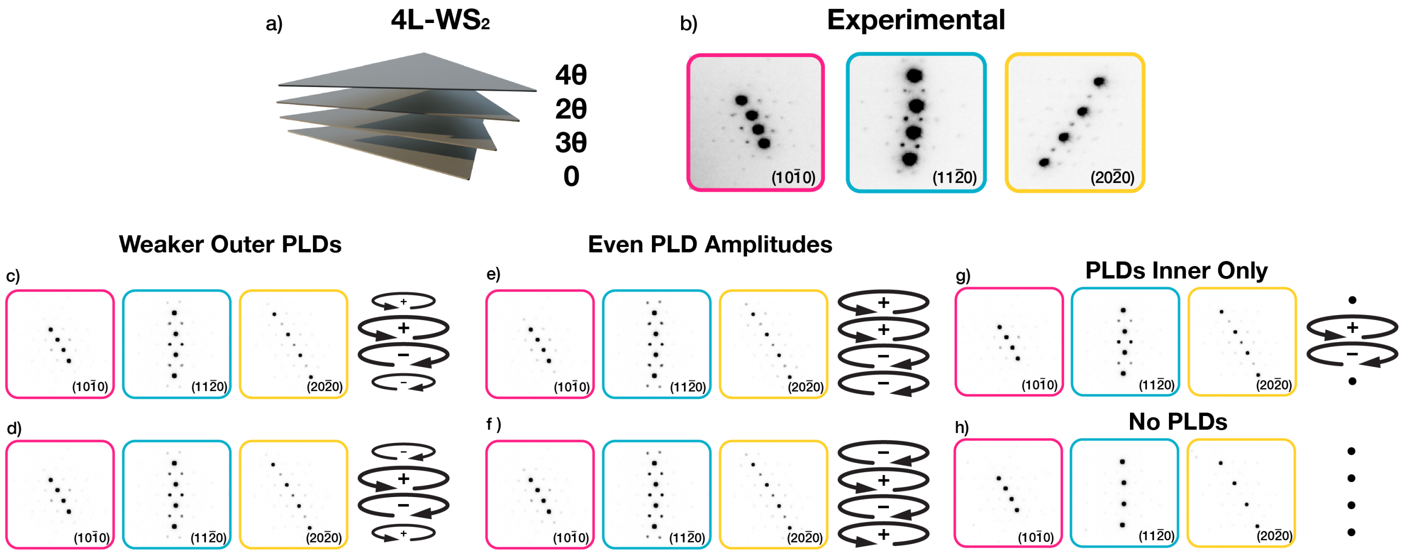

Fig.S11 | Torsional PLDs in 4L-WS_2 a) Schematic diagram of 4L-WS_2 showing twist configuration of each layer. b) Experimental SAED patterns for 4L-WS_2. c–h) Simulated electron diffraction patterns for 4L-WS2 with different torsional PLD amplitudes across layers. Accompanying pictographs denotes sign (+/–) and amplitude of torsional PLDs. c, d) PLD amplitudes are weaker (50%) in outer two layers than in inner layers. e, f) PLD amplitudes are equal in all four layers. g) PLDs exists in inner two layers with no PLD in outer layers. h) Simulations without PLDs. c, e) The sign of PLD in upper two and bottom two layers are equal. d, f) PLD sign alternates. In all cases, superlattice peak intensities are qualitatively similar, while some superlattice peaks are different. Experimental SAED patterns (Fig. 5b) matches closely with c) and d).

Supplementary Figure S12 SAED of $WSe_2/MoSe_2$ Heterostructure

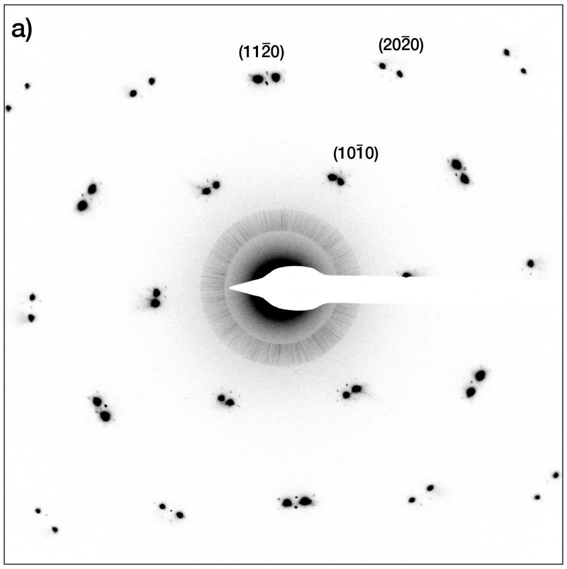

Fig.S12 | SAED of $WSe_2/MoSe_2$ Heterostructure a) SAED pattern of twisted $WSe_2/MoSe_2$ heterostructure shows azimuthally distributed superlattice peaks—characteristics of torsional PLD.

Supplementary Notes 1 Definitions: Twisted Bilayer Graphene and its torsional PLD

Here, we define the real and reciprocal lattice constants (a; $b = \frac{4\pi}{a\sqrt{3}}$) and vectors for unrelaxed twisted bilayer graphene with twist angle θ. The real and reciprocal lattice vectors of constituent layers are defined:

Top Layer	Bottom Layer
$\mathbf{a}_1^t = a\big(\cos(-\frac{\theta}{2})\hat{\mathbf{x}} + \sin(-\frac{\theta}{2})\hat{\mathbf{y}}\big)$	$\mathbf{a}_1^b = a\big(\cos(\frac{\theta}{2})\hat{\mathbf{x}} + \sin(\frac{\theta}{2})\hat{\mathbf{y}}\big)$
$\mathbf{a}_2^t = a\big(\cos(\frac{2\pi}{3} - \frac{\theta}{2})\hat{\mathbf{x}} + \sin(\frac{2\pi}{3} - \frac{\theta}{2})\hat{\mathbf{y}}\big)$	$\mathbf{a}_2^b = a\big(\cos(\frac{2\pi}{3} + \frac{\theta}{2})\hat{\mathbf{x}} + \sin(\frac{2\pi}{3} + \frac{\theta}{2})\hat{\mathbf{y}}\big)$
$\mathbf{b}_1^t = b\big(\cos(\frac{\pi}{6} - \frac{\theta}{2})\hat{\mathbf{x}} + \sin(\frac{\pi}{6} - \frac{\theta}{2})\hat{\mathbf{y}}\big)$	$\mathbf{b}_1^b = b\big(\cos(\frac{\pi}{6} + \frac{\theta}{2})\hat{\mathbf{x}} + \sin(\frac{\pi}{6} + \frac{\theta}{2})\hat{\mathbf{y}}\big)$
$\mathbf{b}_2^t = b\big(\cos(\frac{\pi}{2} - \frac{\theta}{2})\hat{\mathbf{x}} + \sin(\frac{\pi}{2} - \frac{\theta}{2})\hat{\mathbf{y}}\big)$	$\mathbf{b}_2^b = b\big(\cos(\frac{\pi}{2} + \frac{\theta}{2})\hat{\mathbf{x}} + \sin(\frac{\pi}{2} + \frac{\theta}{2})\hat{\mathbf{y}}\big)$

Two layers stacked at an angle θ form a moiré pattern with greater periodicity in real space. The real and reciprocal lattice constants (a_m, q) of the moiré supercell are defined:

$$a_m = \kappa a; \quad q = \frac{4\pi}{a_m\sqrt{3}} = \frac{b}{\kappa} \qquad (1)$$
$$\kappa = \big(2\sin(\frac{\theta}{2})\big)^{-1}$$

κ is a geometrically defined scaling factor of the moiré lattice constant with respect to the crystal lattice constant. The corresponding real and reciprocal lattice vectors of the moiré ($\mathbf{a}_m$, $\mathbf{q}$) are defined to be:

Real Space	Reciprocal Space
$\mathbf{a}_{m1} = a_m\big(\cos(\frac{\pi}{2})\hat{\mathbf{x}} + \sin(\frac{\pi}{2})\hat{\mathbf{y}}\big)$	$\mathbf{b}_{m1} = q\big(\cos(\frac{2\pi}{3})\hat{\mathbf{x}} + \sin(\frac{2\pi}{3})\hat{\mathbf{y}}\big)$
$\mathbf{a}_{m2} = a_m\big(\cos(\frac{7\pi}{6})\hat{\mathbf{x}} + \sin(\frac{7\pi}{6})\hat{\mathbf{y}}\big)$	$\mathbf{b}_{m2} = q\big(\cos(\pi)\hat{\mathbf{x}} + \sin(\pi)\hat{\mathbf{y}}\big)$

The complete torsional PLD field (Δ_{N}) is a sum over each n-th harmonic of the torsional PLD (Δ_n) up to a total of N harmonics. Larger number (N) of Fourier harmonics is needed to accurately depict sharp boundaries at low twist angles.

$$\Delta_N = \sum_n^N \Delta_n \qquad (2)$$

$$\Delta_n = \mathrm{A}_n\big(\hat{\mathbf{A}}_{100}\sin(n\mathbf{q}_{100}\cdot\mathbf{r}) + \hat{\mathbf{A}}_{010}\sin(n\mathbf{q}_{010}\cdot\mathbf{r}) + \hat{\mathbf{A}}_{001}\sin(n\mathbf{q}_{001}\cdot\mathbf{r})\big);\ \hat{\mathbf{A}} \perp \mathbf{q} \qquad (3)$$

Each harmonic Δ_n is a linear superposition of three PLD waves, with the order of harmonic (n) dictating the wave amplitude ($A_n = A_1 e^{-\kappa n}$) and frequency. From the symmetry of real space distortion, We define one of the PLD wave vectors ($\mathbf{q}_{010}$) to be the moiré reciprocal vector $\mathbf{b}_{m2}$ and define $\mathbf{q}_{100}$ and $\mathbf{q}_{001}$ to be $\pm 120°$ away from $\mathbf{q}_{010}$. In addition, for transverse waves in a torsional PLD, displacement vectors ($\hat{\mathbf{A}}$) are defined to be perpendicular to ($\mathbf{q}$):

$\mathbf{q}_{100} = q\big(\cos(\frac{\pi}{3})\hat{\mathbf{x}} + \sin(\frac{\pi}{3})\hat{\mathbf{y}}\big) = q\big(\frac{1}{2}\hat{\mathbf{x}} + \frac{\sqrt{3}}{2}\hat{\mathbf{y}}\big)$	$\hat{\mathbf{A}}_{100} = \big(\cos(\frac{5\pi}{6})\hat{\mathbf{x}} + \sin(\frac{5\pi}{6})\hat{\mathbf{y}}\big) = -\frac{\sqrt{3}}{2}\hat{\mathbf{x}} + \frac{1}{2}\hat{\mathbf{y}}$
$\mathbf{q}_{010} = q\big(\cos(\pi)\hat{\mathbf{x}} + \sin(\pi)\hat{\mathbf{y}}\big) = -q\hat{\mathbf{x}}$	$\hat{\mathbf{A}}_{010} = \big(\cos(\frac{3\pi}{2})\hat{\mathbf{x}} + \sin(\frac{3\pi}{2})\hat{\mathbf{y}}\big) = -\hat{\mathbf{y}}$
$\mathbf{q}_{001} = q\big(\cos(\frac{5\pi}{3})\hat{\mathbf{x}} + \sin(\frac{5\pi}{3})\hat{\mathbf{y}}\big) = q\big(\frac{1}{2}\hat{\mathbf{x}} - \frac{\sqrt{3}}{2}\hat{\mathbf{y}}\big)$	$\hat{\mathbf{A}}_{001} = \big(\cos(\frac{\pi}{6})\hat{\mathbf{x}} + \sin(\frac{\pi}{6})\hat{\mathbf{y}}\big) = \frac{\sqrt{3}}{2}\hat{\mathbf{x}} + \frac{1}{2}\hat{\mathbf{y}}$

Using the definitions above, Δ_n can be expanded:

$$\begin{aligned}\Delta_n = \ & \hat{\mathbf{x}}\big[-\frac{A_n\sqrt{3}}{2}(\sin(n\mathbf{q}_{100}\cdot\mathbf{r}) - \sin(n\mathbf{q}_{001}\cdot\mathbf{r}))\big] \\ & + \hat{\mathbf{y}}\big[\frac{A_n}{2}(\sin(n\mathbf{q}_{100}\cdot\mathbf{r}) + \sin(n\mathbf{q}_{001}\cdot\mathbf{r}) - 2\sin(n\mathbf{q}_{010}\cdot\mathbf{r}))\big]\end{aligned} \qquad (4)$$

For later conveniences, partial derivatives are calculated :

$$\boxed{\begin{aligned}
\frac{\partial\Delta_{n,x}}{\partial x} &= -\tfrac{nqA_n\sqrt{3}}{4}(\cos(n\mathbf{q}_{100}\cdot\mathbf{r}) - \cos(n\mathbf{q}_{001}\cdot\mathbf{r}))\\
\frac{\partial\Delta_{n,y}}{\partial y} &= \tfrac{nqA_n\sqrt{3}}{4}(\cos(n\mathbf{q}_{100}\cdot\mathbf{r}) - \cos(n\mathbf{q}_{001}\cdot\mathbf{r})) = -\frac{\partial\Delta_{n,x}}{\partial x}\\
\frac{\partial\Delta_{n,x}}{\partial y} &= -\tfrac{3nqA_n}{4}(\cos(n\mathbf{q}_{100}\cdot\mathbf{r}) + \cos(n\mathbf{q}_{001}\cdot\mathbf{r}))\\
\frac{\partial\Delta_{n,y}}{\partial x} &= \tfrac{nqA_n}{4}(\cos(n\mathbf{q}_{100}\cdot\mathbf{r}) + \cos(n\mathbf{q}_{001}\cdot\mathbf{r}) + 4\cos(n\mathbf{q}_{010}\cdot\mathbf{r}))
\end{aligned}}$$

Supplementary Notes 2 Derivation: Local Rotation of Torsional PLD

To understand the resulting distortion of a displacement field ($\mathbf{u}$), we calculate the tensor gradient ($\nabla\mathbf{u}$) to obtain the local strain ($\underline{\underline{\epsilon}}$) and rotation ($\underline{\underline{\omega}}$) tensors. We ignore out-of-plane strain and stress for this model.

$$\nabla\mathbf{u} = \begin{bmatrix} \frac{\partial u_x}{\partial x} & \frac{\partial u_x}{\partial y} \\ \frac{\partial u_y}{\partial x} & \frac{\partial u_y}{\partial y} \end{bmatrix} = \underline{\underline{\epsilon}} + \underline{\underline{\omega}} \tag{5}$$

where,

$$\underline{\underline{\epsilon}} = \begin{bmatrix} \frac{\partial u_x}{\partial x} & \frac{1}{2}(\frac{\partial u_x}{\partial y} + \frac{\partial u_y}{\partial x}) \\ \frac{1}{2}(\frac{\partial u_x}{\partial y} + \frac{\partial u_y}{\partial x}) & \frac{\partial u_y}{\partial y} \end{bmatrix} \tag{6}$$

$$\underline{\underline{\omega}} = \begin{bmatrix} 0 & -\frac{1}{2}(\frac{\partial u_y}{\partial x} - \frac{\partial u_x}{\partial y}) \\ \frac{1}{2}(\frac{\partial u_y}{\partial x} - \frac{\partial u_x}{\partial y}) & 0 \end{bmatrix} \tag{7}$$

Here, we focus on $\underline{\underline{\omega}}$—also known as the infinitesimal rotational tensor—that describes local rotation due to the displacement field $\mathbf{u}$. We define the non-zero off-diagonal component of $\underline{\underline{\omega}}$ as rotational field Ω that characterizes the local twist due to $\mathbf{u}$. Notably, Ω is proportional to the curl of displacement field ($\nabla\times\mathbf{u}$).

$$\underline{\underline{\omega}} = \begin{bmatrix} 0 & -\Omega \\ \Omega & 0 \end{bmatrix} = \begin{bmatrix} 0 & -\frac{1}{2}(\frac{\partial u_y}{\partial x} - \frac{\partial u_x}{\partial y}) \\ \frac{1}{2}(\frac{\partial u_y}{\partial x} - \frac{\partial u_x}{\partial y}) & 0 \end{bmatrix} \tag{8}$$

From the definitions above, the rotational field due to Torsional PLD of nth harmonic (Ω_n) is :

$$\begin{aligned}
\Omega_n &= \frac{1}{2}(\frac{\partial\Delta_{n,y}}{\partial x} - \frac{\partial\Delta_{n,x}}{\partial y}) \\
&= \frac{nqA_n}{8}\Big(3(\cos(n\mathbf{q}_{100}\cdot\mathbf{r}) + \cos(n\mathbf{q}_{001}\cdot\mathbf{r})) + (\cos(n\mathbf{q}_{100}\cdot\mathbf{r}) + 4\cos(n\mathbf{q}_{010}\cdot\mathbf{r}) + \cos(n\mathbf{q}_{001}\cdot\mathbf{r}))\Big) \\
&= \frac{nqA_n}{2}\Big(\cos(n\mathbf{q}_{100}\cdot\mathbf{r}) + \cos(n\mathbf{q}_{010}\cdot\mathbf{r}) + \cos(n\mathbf{q}_{001}\cdot\mathbf{r})\Big)
\end{aligned} \tag{9}$$

The local rotation at AA and AB core, $\mathbf{r}_{AA} = 0$ and $\mathbf{r}_{AB} = \frac{a_m}{\sqrt{3}}\hat{\mathbf{x}}$, are:

$$\Omega_n(\mathbf{r}_{AA}) = \frac{3nqA_n}{2}, \qquad \Omega_n(\mathbf{r}_{AB}) = \begin{cases} \frac{3nqA_n}{2} & n/3 \in \mathbb{Z} \\ -\frac{3nqA_n}{4} & else \end{cases} \tag{10}$$

Therefore, rotation in AA core is twice stronger than and opposite to that of AB core for the fundamental frequency (A_1).

The total rotational field due to the PLD is trivially ($\Omega_{\mathrm{N}} = \sum_n^N \Omega_n$), as differentiation and summation commutes. Figure S7 depicts Ω_{N} for N=1, 3, and 7 with $A_n = A_1 e^{-\kappa n}$.

Supplementary Notes 2.1 Geometric Upper Bound for PLD Amplitude (A_1)

We are interested in the PLD field near a AB core that is dominated by the fundamental PLD harmonic (n=1). The local rotation there is:

$$\Omega_1(\mathbf{r}_{AB}) = -\frac{3qA_1}{4} \tag{11}$$

For torsional PLD in each layer to locally restore AB cores, torsional PLD needs to locally twist the AB cores by $\frac{\theta}{2}$:

$$\begin{aligned} -\frac{3qA_1}{4} &= \frac{\theta}{2} \\ \mathrm{A}_1 &= -\frac{4\theta}{6q}; \quad q = \frac{4\pi}{a\sqrt{3}}(2\sin(\frac{\theta}{2})) \approx \frac{4\pi\theta}{a\sqrt{3}} \\ \mathrm{A}_1 &= -\frac{a}{2\pi\sqrt{3}} \end{aligned} \tag{12}$$

This defines the upper bound for torsional PLD amplitude. Notably, the upper bound is twist angle independent. For graphene ($a = 2.46$Å), $\mathrm{A}_1 = 22.6$ pm is the upper bound.

Supplementary Notes 3 Derivation: Stress, Strain and Elastic Energy of Torsional PLD

To calculate the strain tensor, ($\underline{\underline{\epsilon}}$), we first consider a strain tensor from the nth harmonic of torsional PLD ($\underline{\underline{\epsilon_n}}$).

$$\underline{\underline{\epsilon_n}} = \begin{bmatrix} \epsilon_{n,xx} & \epsilon_{n,xy} \\ \epsilon_{nxy} & \epsilon_{n,yy} \end{bmatrix} = \begin{bmatrix} \frac{\partial \Delta_{n,x}}{\partial x} & \frac{1}{2}(\frac{\partial \Delta_{n,x}}{\partial y} + \frac{\partial \Delta_{n,y}}{\partial x}) \\ \frac{1}{2}(\frac{\partial \Delta_{n,x}}{\partial y} + \frac{\partial \Delta_{n,y}}{\partial x}) & \frac{\partial \Delta_{n,y}}{\partial y} \end{bmatrix} \tag{13}$$

.

$$\epsilon_{n,xx} = \frac{\partial \Delta_{n,x}}{\partial x} = -\frac{nqA_n\sqrt{3}}{4}(\cos(n\mathbf{q}_{100}\cdot\mathbf{r}) - \cos(n\mathbf{q}_{001}\cdot\mathbf{r})) \tag{14}$$

$$\epsilon_{n,yy} = \frac{\partial \Delta_{n,y}}{\partial y} = -\epsilon_{xx} \tag{15}$$

$$\begin{aligned} \epsilon_{n,xy} &= \frac{1}{2}(\frac{\partial \Delta_{n,x}}{\partial y} + \frac{\partial \Delta_{n,y}}{\partial x}) \\ &= -\frac{nqA_n}{4}(\cos(n\mathbf{q}_{100}\cdot\mathbf{r}) + \cos(n\mathbf{q}_{001}\cdot\mathbf{r}) - 2\cos(n\mathbf{q}_{010}\cdot\mathbf{r})) \end{aligned} \tag{16}$$

Total strain is

$$\underline{\underline{\epsilon}} = \sum_n^N \underline{\underline{\epsilon_n}}; \qquad \epsilon_{ij} = \sum_n^N \epsilon_{n,ij} \tag{17}$$

Supplementary Notes 3.1 Stiffness Tensor, Stress and Elastic Energy

As the torsional PLD here deals with in-plane distortions, and graphene is an isotopic material, we assume plane stress deformation with the stress-strain relationship as follows:

$$\begin{aligned} \begin{pmatrix} \sigma_{xx} \\ \sigma_{yy} \\ \sigma_{xy} \end{pmatrix} &= \frac{E}{1-\nu^2} \begin{bmatrix} 1 & \nu & 0 \\ \nu & 1 & 0 \\ 0 & 0 & \frac{1-\nu}{2} \end{bmatrix} \begin{pmatrix} \epsilon_{xx} \\ \epsilon_{yy} \\ 2\epsilon_{xy} \end{pmatrix} \\ &= \frac{2G}{1-\nu} \begin{bmatrix} 1 & \nu & 0 \\ \nu & 1 & 0 \\ 0 & 0 & \frac{1-\nu}{2} \end{bmatrix} \begin{pmatrix} \epsilon_{xx} \\ \epsilon_{yy} \\ 2\epsilon_{xy} \end{pmatrix} \\ &= \begin{pmatrix} \frac{2G}{1-\nu}(\epsilon_{xx} + \nu\epsilon_{yy}) \\ \frac{2G}{1-\nu}(\nu\epsilon_{xx} + \epsilon_{yy}) \\ 2G\epsilon_{xy} \end{pmatrix} \end{aligned} \tag{18}$$

E, G, ν are Young's modulus, Shear modulus and Poisson ratio respectively. We used the usual $E = 2G(1+\nu)$ relationship. The local elastic energy (v_{El}), assuming Hookean elasticity, is defined as:

$$\begin{aligned} v_{El} &= \frac{1}{2}\sigma_{xx}\epsilon_{xx} + \frac{1}{2}\sigma_{yy}\epsilon_{yy} + \sigma_{xy}\epsilon_{xy} \\ &= \frac{G}{1-\nu}(\epsilon_{xx}^2 + \epsilon_{yy}^2 + 2\nu\epsilon_{xx}\epsilon_{yy}) + 2G\epsilon_{xy}^2 \\ &= \frac{G}{1-\nu}((\epsilon_{xx} + \epsilon_{yy})^2 - 2(1-\nu)\epsilon_{xx}\epsilon_{yy}) + 2G\epsilon_{xy}^2 \\ &= \frac{G}{1-\nu}(\epsilon_{xx} + \epsilon_{yy})^2 + 2G(\epsilon_{xy}^2 - \epsilon_{xx}\epsilon_{yy}) \\ &= 2G(\epsilon_{xx}^2 + \epsilon_{xy}^2) \qquad \because \epsilon_{xx} = -\epsilon_{yy} \text{ for Torsional PLD} \end{aligned} \tag{19}$$

Supplementary Notes 3.2 Elastic Energy per Unit Cell

The elastic energy term (v_{El}) is related to the spatial derivatives of torsional PLD field ($\frac{\partial \Delta_{n,i}}{\partial i}$) and describes the infinitesimal elastic energy at particular lattice positions. To obtain the elastic energy per moiré unit cell (V_{El}), we integrate v_{El} over the spatial dimensions of a moiré unit cell, μ.

$$\begin{aligned} V_{El} &= \iint_\mu dydx\; v_{El} = 2G \iint_\mu dydx\; (\epsilon_{xx}^2 + \epsilon_{xy}^2) \\ &= 2G \iint_\mu dydx\; [(\sum_n^N \epsilon_{n,xx})^2 + (\sum_n^N \epsilon_{n,xy})^2] \\ &= 2G \sum_n^N \iint_\mu dydx\; \left[(\epsilon_{n,xx}^2 + \epsilon_{n,xy}^2)\right] + 2G \sum_n^N \sum_{m\neq n}^N \iint_\mu dydx\; \left[(\epsilon_{n,xx}\epsilon_{m,xx} + \epsilon_{n,xy}\epsilon_{m,xy})\right] \end{aligned} \tag{20}$$

Substituting values from equation (14) – (16):

$$\begin{aligned} \iint_\mu dydx\; (\epsilon_{n,xx}^2 + \epsilon_{n,xy}^2) &= \frac{3n^2q^2A_n^2}{4} \iint_\mu dydx\; \left[\cos^2(n\mathbf{q}_{010}\cdot\mathbf{r}) + \cos(n\mathbf{q}_{100}\cdot\mathbf{r})\cos(n\mathbf{q}_{001}\cdot\mathbf{r})\right] \\ &= \frac{3n^2q^2A_n^2}{4} \int_0^{a_m} dy \int_0^{\frac{a_m\sqrt{3}}{2}} dx\; \left[\cos^2(nqx) + \frac{1}{2}(\cos(nqx)\cos(nq\sqrt{3}y))\right] \\ &= \frac{3n^2q^2A_n^2}{4n^2q^2\sqrt{3}}(4\pi n)(\pi n) = \sqrt{3}n^2\pi^2A_n^2 \end{aligned} \tag{21}$$

$$\begin{aligned} &(\epsilon_{n,xx}\epsilon_{m,xx} + \epsilon_{n,xy}\epsilon_{m,xy}) \\ &= \frac{3nmq^2A_n^2}{8}\left[2\cos(n\mathbf{q}_{010}\cdot\mathbf{r})\cos(m\mathbf{q}_{010}\cdot\mathbf{r}) - \cos(n\mathbf{q}_{010}\cdot\mathbf{r})(\cos(m\mathbf{q}_{100}\cdot\mathbf{r}) + \cos(m\mathbf{q}_{001}\cdot\mathbf{r}))\right] \end{aligned} \tag{22}$$

$$\iint_\mu dydx\;\; \cos(n\mathbf{q}_{010}\cdot\mathbf{r})\cos(m\mathbf{q}_{010}\cdot\mathbf{r}) = 0 \because \text{Orthogonality} \tag{23}$$

$$\begin{aligned} &\iint_\mu dydx\;\; \cos(n\mathbf{q}_{010}\cdot\mathbf{r})(\cos(m\mathbf{q}_{100}\cdot\mathbf{r}) + \cos(m\mathbf{q}_{001}\cdot\mathbf{r})) \\ &= \int_0^{a_m} dy \int_0^{\frac{a_m\sqrt{3}}{2}} dx\; 2\cos(nqx)\cos(\frac{mq}{2}x)\cos(\frac{mq\sqrt{3}}{2}y)) \\ &\qquad v = \frac{mq\sqrt{3}y}{2} \\ &= \int_0^{a_m} dy\; 2\cos(nqx)\cos(\frac{mq}{2}x) \int_0^{2\pi m} dv\cos(v)) = 0 \end{aligned} \tag{24}$$

Therefore, all cross-terms are zero:

$$\iint_\mu dydx\; \left[(\epsilon_{n,xx}\epsilon_{m,xx} + \epsilon_{n,xy}\epsilon_{m,xy})\right] = 0 \tag{25}$$

Finally, we calculate the total elastic energy per moirè unit cell in each layer by summing over contributions from a total of N harmonics:

$$V_{El} = 2\sqrt{3}\pi^2 G \sum_n^N n^2 A_n^2 \tag{26}$$

As defined previously, $A_n = A_1 e^{-\kappa n}$. Elastic energy cost of nth harmonic torsional PLD is independent of other harmonics.
